# Intraperitoneally Retained Contraceptive Device After Uterine Perforation: A Case Report

**DOI:** 10.34763/jmotherandchild.20232701.d-22-00054

**Published:** 2023-07-06

**Authors:** Hanan S. Al-Khatlan, Aliaa M. Al-Tuhoo, Mohannad Abu-Faza, Mariam Obaid, Ibrahim A. Abdelazim, Ibrahim M. Al-Kandari

**Affiliations:** Department of Obstetrics and Gynecology, Ahmadi Hospital, Kuwait Oil Company (KOC), Ahmadi, Kuwait; Department of General and Laparoscopic Surgery, Ahmadi Hospital, Kuwait Oil Company (KOC), Ahmadi, Kuwait

**Keywords:** Retained, Peritoneal, Contraceptive Device, Uterine, Perforation

## Abstract

A 29-year-old parous woman with a history of a T-shaped copper intrauterine device (IUD) insertion presented 8 months later with a complaint of the contraceptive device being missing. Computed tomography with contrast turned out to be superior to the combined abdominal and pelvic X-ray and transvaginal ultrasound in providing the detailed extrauterine location of the device between the urinary bladder and uterus. A laparoscopy was successful in the atraumatic freeing of the IUD from omental and bladder adhesions, and in its final removal.

## Introduction

The intrauterine device (IUD) belongs to reversible long-term contraceptive methods. The IUD is an effective method of contraception with generally minimal and tolerable side effects [1,2,3]. However, a wide array of complications associated with the IUD insertion procedure and its intrauterine presence have been described. They include abdominal/back pain, pelvic inflammatory disease, expulsion, and uterine perforation [4]. The latter can occur as an immediate traumatic perforation during IUD insertion or as a late secondary perforation which arises from a slow penetration of the myometrium by the device over a prolonged time.

Overall, uterine perforation during IUD insertion is uncommon, with an incidence ranging from 0.4 to 1.6 per 1,000 insertions [5]. After the perforation, the IUD may stay inside the peritoneal cavity or migrate into any nearby intraabdominal structure [2,3].

The aim of this report is to present a laparoscopic removal of an intraperitoneally located IUD after asymptomatic uterine perforation. Moreover, we offer a brief account of the topic.

## Case Report

A 29-year-old woman, para 2 (2 previous vaginal deliveries at term), with a history of a T-shaped copper IUD insertion in December 2021, presented 8 months later with a complaint of the missing contraceptive device (strings missing in the vagina). The IUD insertion had been seemingly uneventful, as with the uterine perforation being asymptomatic; the patient had not experienced pain nor discomfort.

**Figure 1.**
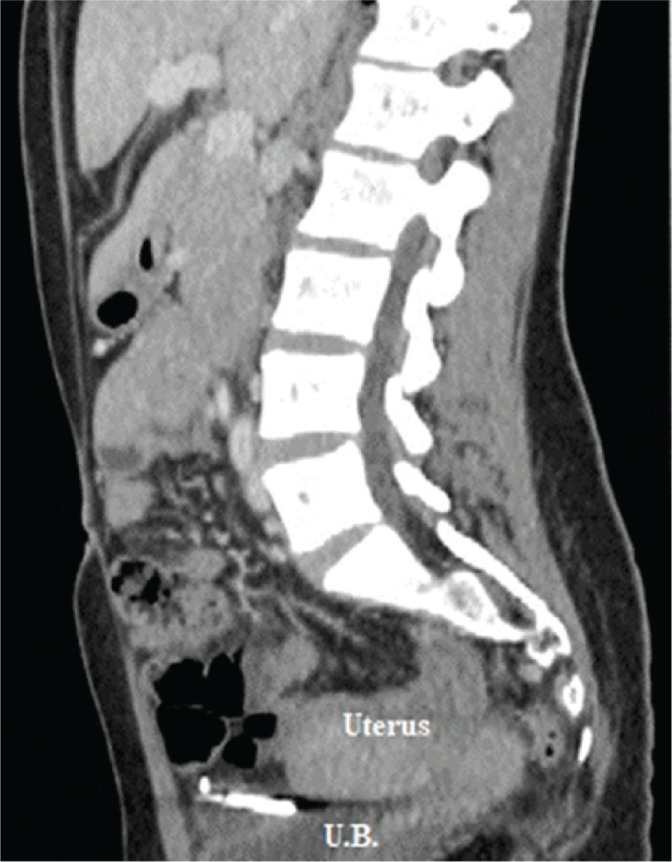
Computed tomography confirms an IUD to be extrauterine in location, between the urinary bladder and uterus.

After the exclusion of pregnancy with human chorionic gonadotropin measurement, both a combined abdominal and pelvic X-ray and a departmental transvaginal ultrasound suggested an extrauterine location of the device. Computed tomography with contrast demonstrated the IUD to be located intraperitoneally between the urinary bladder and uterus (Figure 1).

After obtaining informed consent from the patient and following a preoperative work-up according to the hospital's protocol, she was scheduled for a diagnostic laparoscopy.

**Figure 2.**
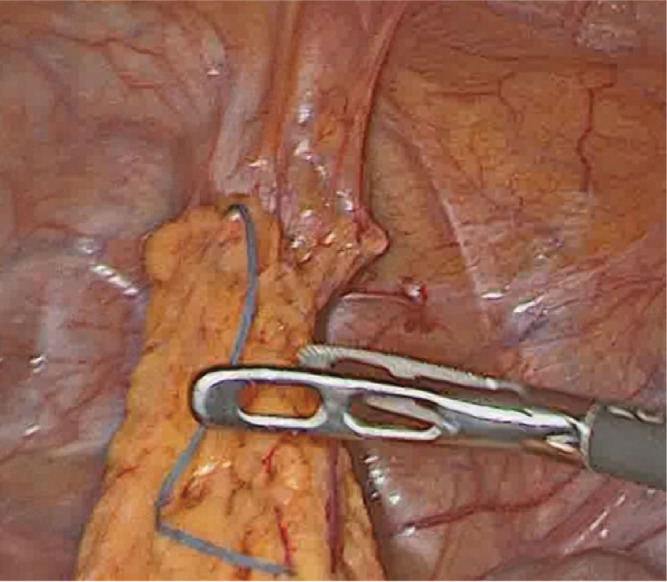
At laparoscopy, the contraceptive device was found to be fully covered by omental adhesions and adherent to the urinary bladder’s dome and superior surface.

**Figure 3.**
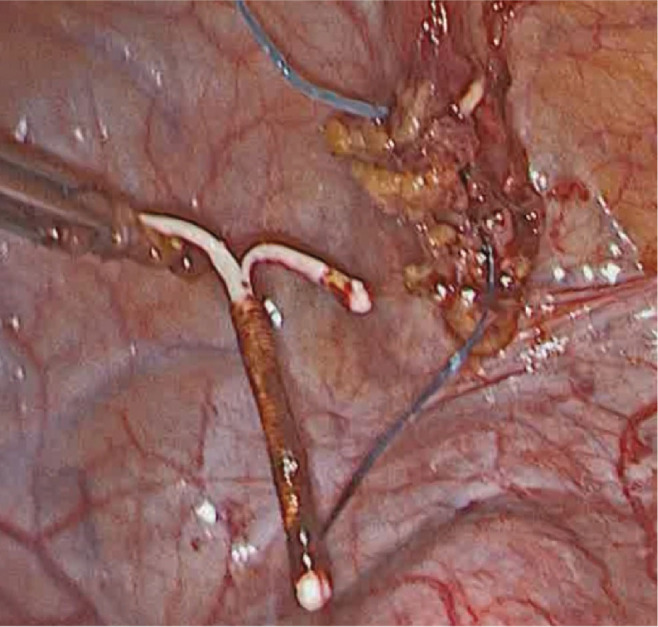
Atraumatic laparoscopic freeing of the IUD from omental and urinary bladder adhesions.

At surgery, the uterus was found to be intact (no visible evidence of perforation). The IUD turned out to be localized intraperitoneally, adherent to the urinary bladder dome and its superior surface by omental adhesions (Fig. 2). This location was in line with the findings from the computed tomography. A step-by-step atraumatic laparoscopic freeing of the device from the omental and urinary bladder adhesions enabled its final removal (Fig. 3). No blood loss was observed. The patient was discharged on the second postoperative day in a good general condition.

## Discussion

Uterine perforation during IUD insertion is believed to occur following forced insertion against the uterine wall [5]. Complete uterine perforation was defined by Zakin et al. as the passing of the IUD through the endometrium, myometrium, and uterine serosa to the peritoneal cavity [6]. Less frequently, the IUD may penetrate the uterus partially and remain within the myometrium (partial perforation) [5].

Uterine perforation is an uncommon complication of IUD insertion; it is often asymptomatic and can pass unnoticed [5]. The risk factors for uterine perforation during IUD placement include insertion by a less experienced physician and postpartum insertion defined as ≤6 months after delivery [5,7].

Transvaginal ultrasounds are considered to be a better modality for identifying intrauterine devices than intraabdominal ones [5]. All IUDs are radiopaque at either their intrauterine or extrauterine location, and can be easily visualized on abdominal and pelvic X-rays, but computed tomography or magnetic resonance imaging may be required for establishing the precise location [5,8], as was the case for our patient.

Some authors have suggested that removal of intraabdominally displaced IUDs is not warranted and that the risk of surgery (either laparoscopy or laparotomy) is not justified, especially in asymptomatic women [5]. The question remains whether the copper IUD components (polyethylene frame, polyethylene threads, and copper ions) can induce peritoneal adhesions or not. In our case study, the device was found adherent to the urinary bladder dome and its superior surface by omental adhesions. Other studies have also shown local peritoneal adhesions around displaced IUDs [9]. Laparoscopic removal of such devices is preferred [6,10]. However, a systematic review found that laparoscopic removal can be successful in as little as 64% of cases [10].

Myometrial healing after perforation is usually rapid, and laparoscopies done a few days or weeks after the perforation note no visible scar in most cases [5]. Zakin et al. state that the scar usually disappears within 2 months [6], which is in line with the lack of apparent scarring on the uterus of our patient.

## Conclusions

Uterine perforations associated with contraceptive device insertion are mainly immediate traumatic perforations. Such situations are usually asymptomatic, and such, are frequently due to the intraabdominal presence of the device, even if surrounded by local peritoneal adhesions. Although transvaginal ultrasounds and combined abdominal and pelvic X-rays are both effective modalities for identifying the extrauterine location of IUD, in this study, a computed tomography turned out to be superior. The intraperitoneally located contraceptive device should be removed in these cases, and laparoscopy is the preferred route of removal.
